# Rare Direct Locoregional Brain Metastasis and Recurrence in Laryngeal Squamous Cell Carcinoma

**DOI:** 10.7759/cureus.46676

**Published:** 2023-10-08

**Authors:** Moutaz Ghrewati, Anas Mahmoud, Tala Beilani, Mehandar Kumar

**Affiliations:** 1 Hematology and Oncology, St. Joseph's Regional Medical Center, Paterson, USA; 2 Internal Medicine, St. Joseph’s University Medical Center, Paterson, USA; 3 Oncology, Kansas City University, Kansas City, USA

**Keywords:** chemotherapy, head and neck squamous cell carcinoma (hnscc), laryngeal squamous cell carcinomas (lsccs), perineural invasion (pni), radiation therapy, total laryngectomy

## Abstract

Laryngeal cancer is predominantly a squamous cell in origin that can present with voice changes and difficulty or pain with swallowing. It is more likely to cause local spread than distant ones. The prognosis depends on multiple factors, including the stage, tumor differentiation, extranodal extension, and adjuvant therapy. Head and neck cancers have a higher tendency for perineural invasion and spread, one of the most vital factors correlating with poor outcomes and recurrence rates. We present a rare case of a 52-year-old male with an extensive history of tobacco use (five packs per day over 30 years) who developed laryngeal squamous cell carcinoma that spread to the brain despite total laryngectomy and adjuvant radiation therapy. Despite resection of the brain metastasis, the tumor metastasized again in the brain through perineural spread. Due to the side effects of repeated radiotherapy and starting chemotherapy, the patient opted for comfort care and refused further treatment. The perineural spread of head and neck cancers is not abundant in the literature, and we believe our case will add to the future treatment of head and neck cancers with perineural invasion.

## Introduction

Head and neck squamous cell carcinoma (HNSCC) can arise from squamous cells lining the oral cavity, oro/hypopharynx, larynx, and nasopharynx. Smoking and alcohol abuse are the most common risk factors, followed by high-risk human papillomavirus (HPV). Worldwide, 644,000 new HNSCC cases each year are reported [1]. Of all HNSCC cases, 184615 are laryngeal squamous cell carcinoma (LSCC), with 99840 deaths in 2020 [2]. LSCC mainly affects the glottis in 70% of the cases, followed by the supraglottis and subglottis. HNSCC has a local spread tendency to regional lymph nodes, and around 10% can cause distant metastasis [3]. As low as two percent, HNSCC can cause brain metastasis [BM] [4], and the laryngeal origin of HNSCC is far less common [5]. Hence, one must apply the American Joint Committee on Cancer's TNM staging system for glottic cancer to select the most appropriate treatment option [6]. Nonetheless, managing LSCC with perineural spread to the brain is now well established in recurrent cases. Although radiotherapy has shown benefits in treating LSCC with BM, repeat radiotherapy carries a high risk of tissue necrosis. The development of chemotherapy and immunotherapy for the locoregional recurrence of LSCC represents the future line of treatment for these challenging cases. We present a patient who developed LSCC with perineural invasion to the brain, which was treated surgically and with adjuvant radiotherapy; however, LSCC had a locoregional recurrence, which made the treatment very difficult to treat. Very little data has been published about the locoregional recurrence of LSCC with perineural invasion of the brain, and we hope our case can benefit the future management of these rare cases. 

## Case presentation

**Figure 1:**
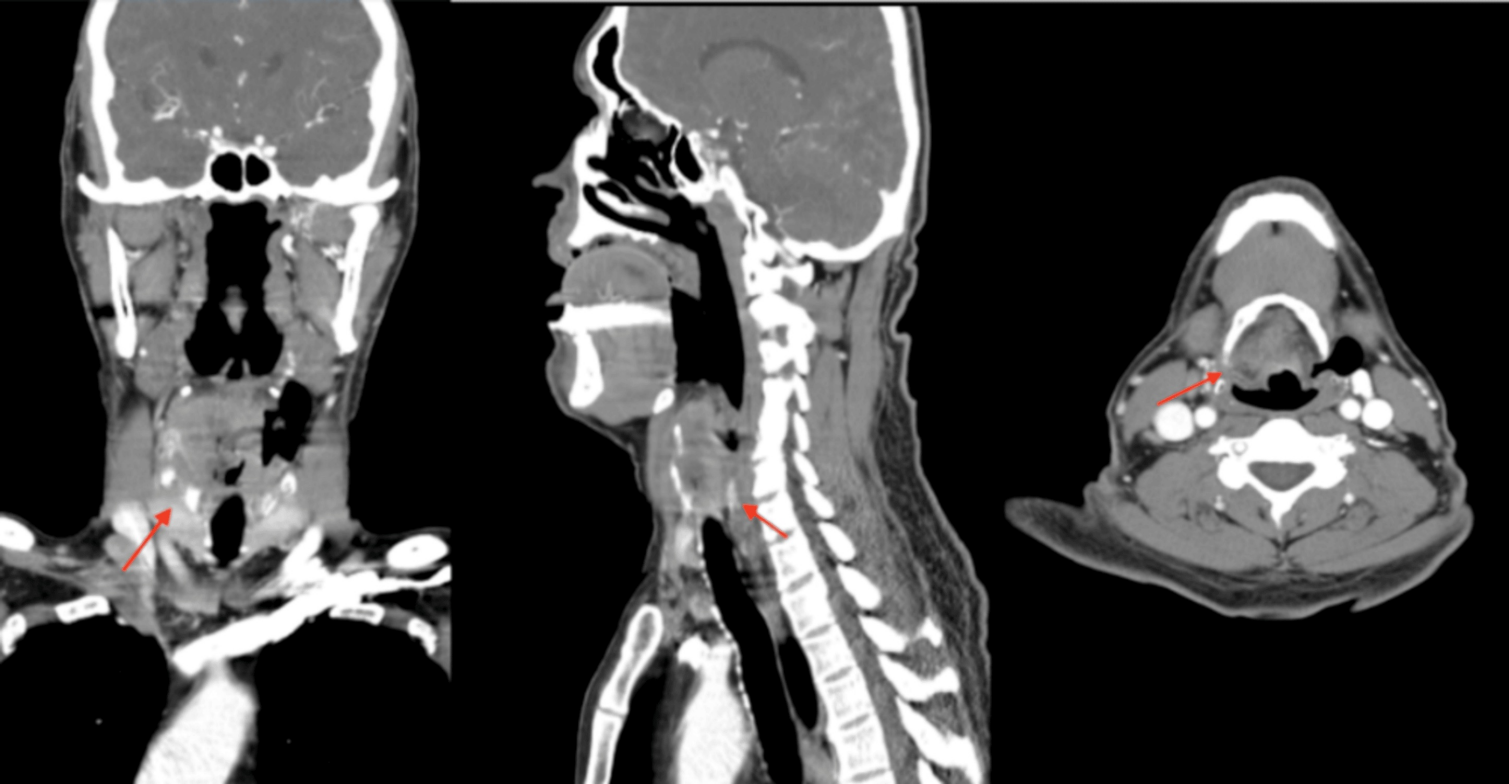
CT scan of the head and neck (multiple views) A CT scan of the head and neck shows supraglottic mass extending to the vocal cords and appears to be extending around the lateral aspect of the true vocal cords, with some narrowing of the vocal cord level (red lines).

Our patient is a 52-year-old male with a past medical history of tobacco use who presented to the emergency department (ED) complaining of the hoarseness of his voice over the last week. The patient reported that he has been having shortness of breath and a sore throat over the last three months, which were gradually progressing and were later accompanied by hoarseness in his voice. Also, he noticed a 14-pound weight loss over the same period. He denied fever, chest pain, cough, recent infections, or other constitutional symptoms. Vitally, the patient was stable, as his blood pressure was 130/70, his heart rate was 80, his oxygen saturation was 97% on room air, and his temperature was 97 °F. Blood lab results were within normal limits. A computerized tomography (CT) scan of the neck showed a supraglottic mass extending to the vocal cords and appears to be extending around the lateral aspect of the true vocal cords, with some narrowing of the vocal cord level (Figure 1). Ear, nose, and throat (ENT) recommended securing the airway with an awake tracheostomy due to the anticipated imminent respiratory failure secondary to the mass. The patient had an awake tracheostomy in the operating room (OR), and a biopsy was taken using direct laryngoscopy, which revealed an invasive squamous cell carcinoma. The patient then got a prophylactic PEG tube placement and was scheduled for a positron emission tomography (PET) scan, which showed a hypermetabolic mass in the neck without any evidence of distant metastatic disease. The oncologist declared it stage cT4aN1M0.

**Figure 2:**
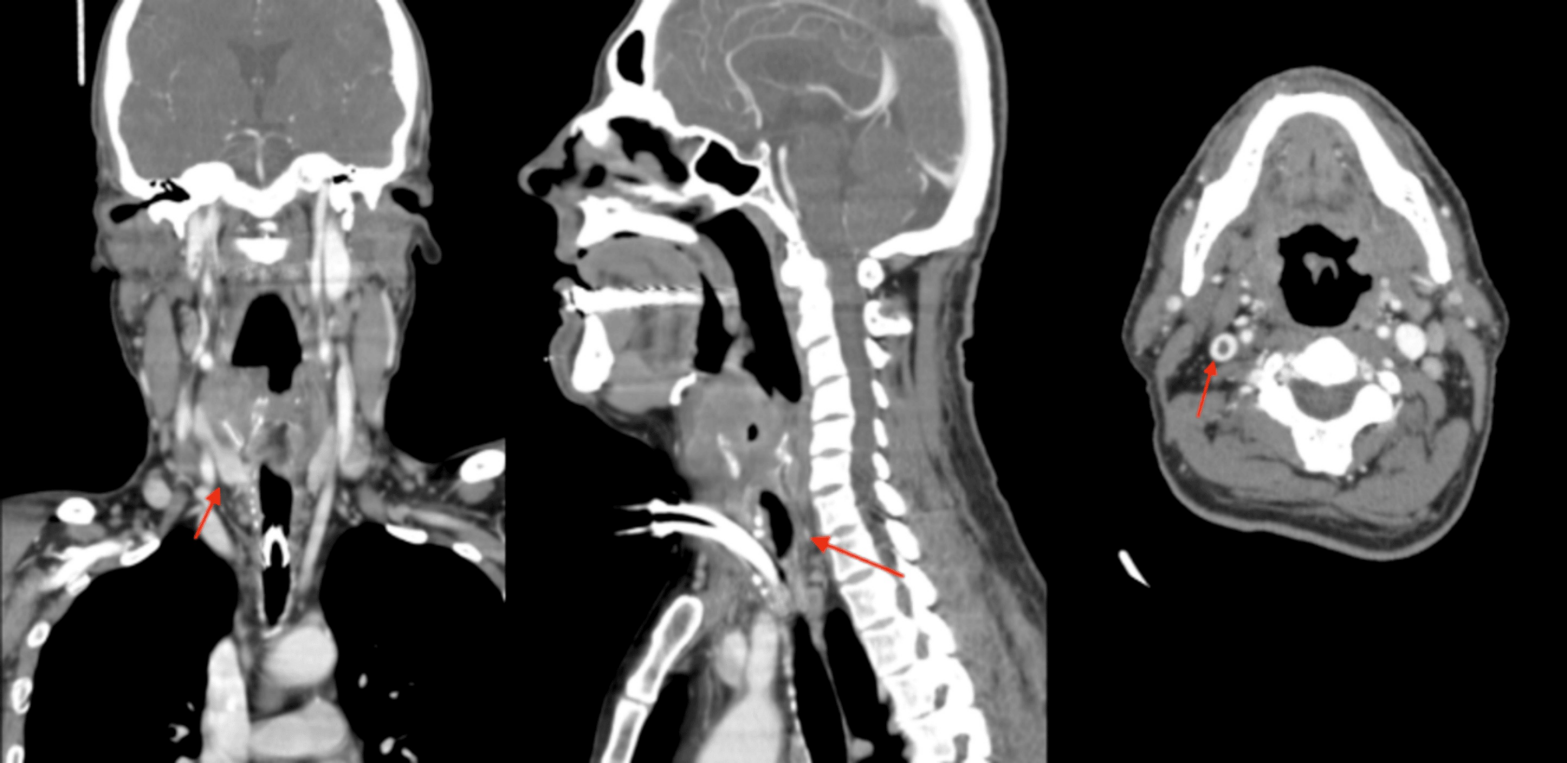
CT scan of the head and neck (multiple views) CT scans of the head and neck show bulky laryngeal mass with transglottic extension and invasion of the thyroid cartilage [red line, left two images], as well as thrombosis of the internal jugular vein (red line, right image).

**Figure 3:**
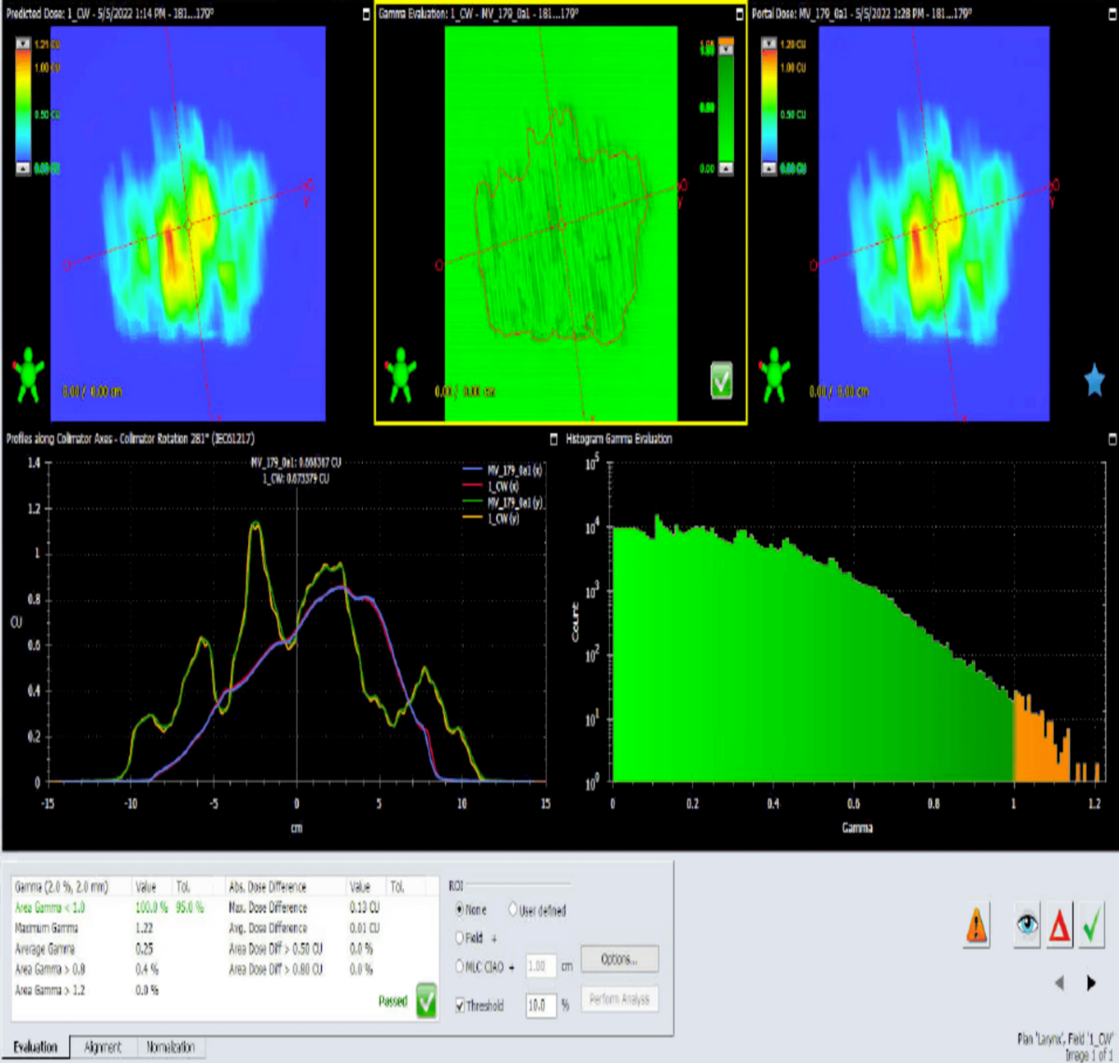
Radiation therapy map Radiation therapy map for the area after laryngectomy and the lymph node.

Unfortunately, before starting treatment, the patient and his family decided not to pursue any treatment initially. However, the patient returned to the emergency department two months later for worsening symptoms. A CT scan of the neck showed a bulky laryngeal mass with transglottic extension, invasion of the thyroid cartilage, and thrombosis of the internal jugular vein (Figure 2). Fortunately, the repeat staging workup did not show any significant disease progression. The patient underwent definitive surgery with a laryngectomy and bilateral neck dissections. Pathology from the operation revealed a 5.3 cm x 5 cm x 4.5 cm squamous cell carcinoma, non-keratinizing, originating in the supraglottis involving the glottis, with no perineural invasion, and a 1/72 lymph node showing metastatic carcinoma. All the margins of resection were negative for carcinoma. The patient started adjuvant radiation therapy to treat the primary and level II nodes. The total dose was 6600 cGy (dose per fraction 220 and number of fractions 30), which the patient completed and improved his health overall significantly. The map therapy is shown in Figure 3 [3].

**Figure 4:**
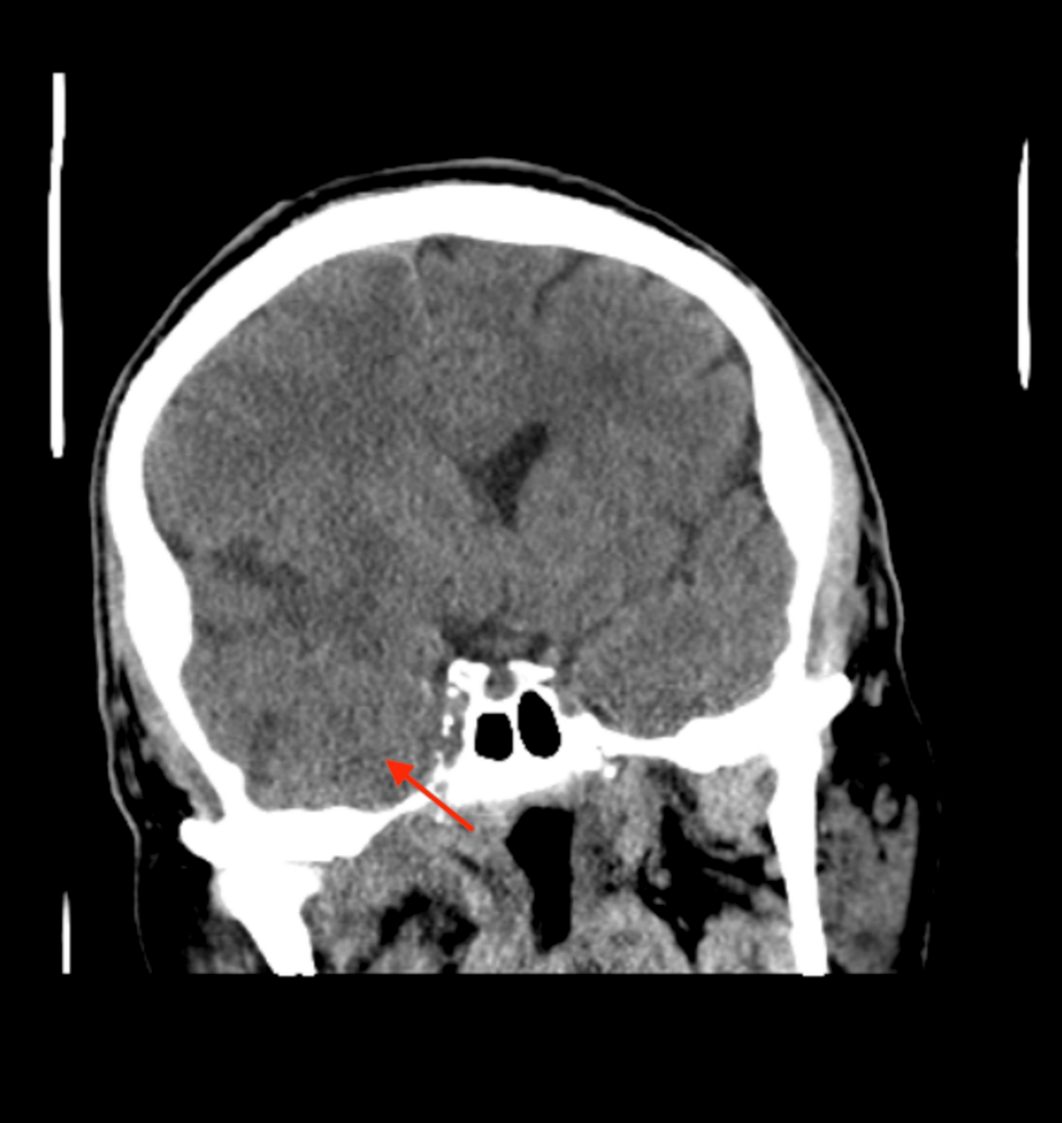
CT scan of the head (coronal section) A CT scan of the head (coronal section) showed cerebral edema in the right frontal and temporal lobes (red arrow), suggesting a possible underlying metastatic process with mass effect.

**Figure 5:**
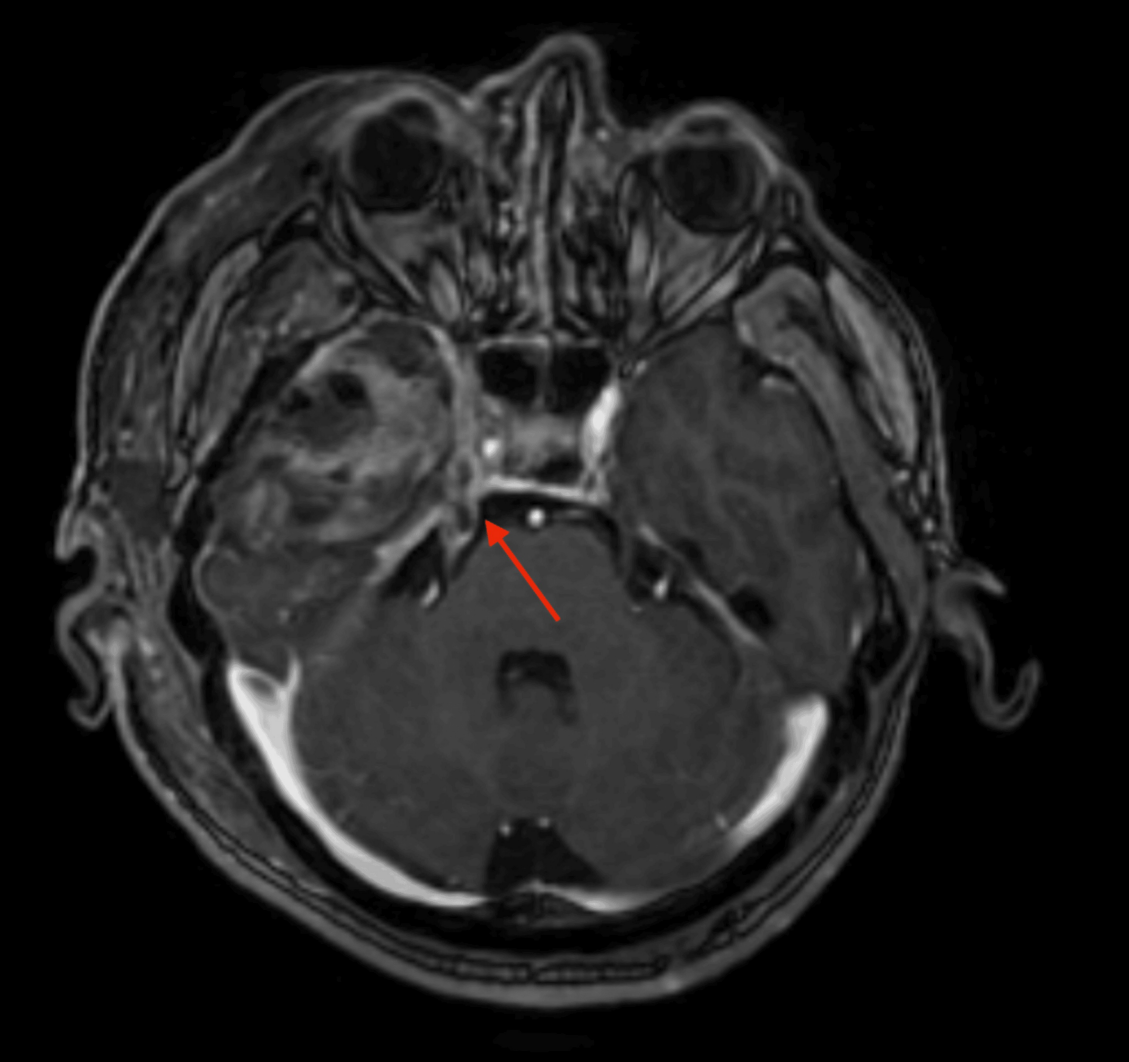
MRI of the brain MRI of the brain shows extensive perineural spread of the tumor through the third division of the trigeminal nerve (red arrow).

Eight months later, the patient presented to the ED with a severe headache, localized to the front, 10/10 in intensity, and not relieved by analgesics. On further questioning, the patient reported he developed mild facial paralysis three months ago without any other neurological symptoms, which he ignored and did not seek any doctor's opinion. A CT scan of the head showed cerebral edema in the right frontal and temporal lobes, suggesting a possible underlying metastatic process with mass effect (Figure 4). Neurosurgery performed a right craniotomy with resection of the right temporal mass. A biopsy confirmed metastatic basaloid squamous cell carcinoma. An MRI of the brain s/p craniotomy showed the extensive perineural spread of the tumor through the third division of the trigeminal nerve (Figure 5). The oncologist reviewed the treatment options with the patient, and the patient chose to proceed with chemotherapy after discussing the case at the tumor board in light of the rarity of locoregional recurrence of laryngeal SCC.

**Figure 6:**
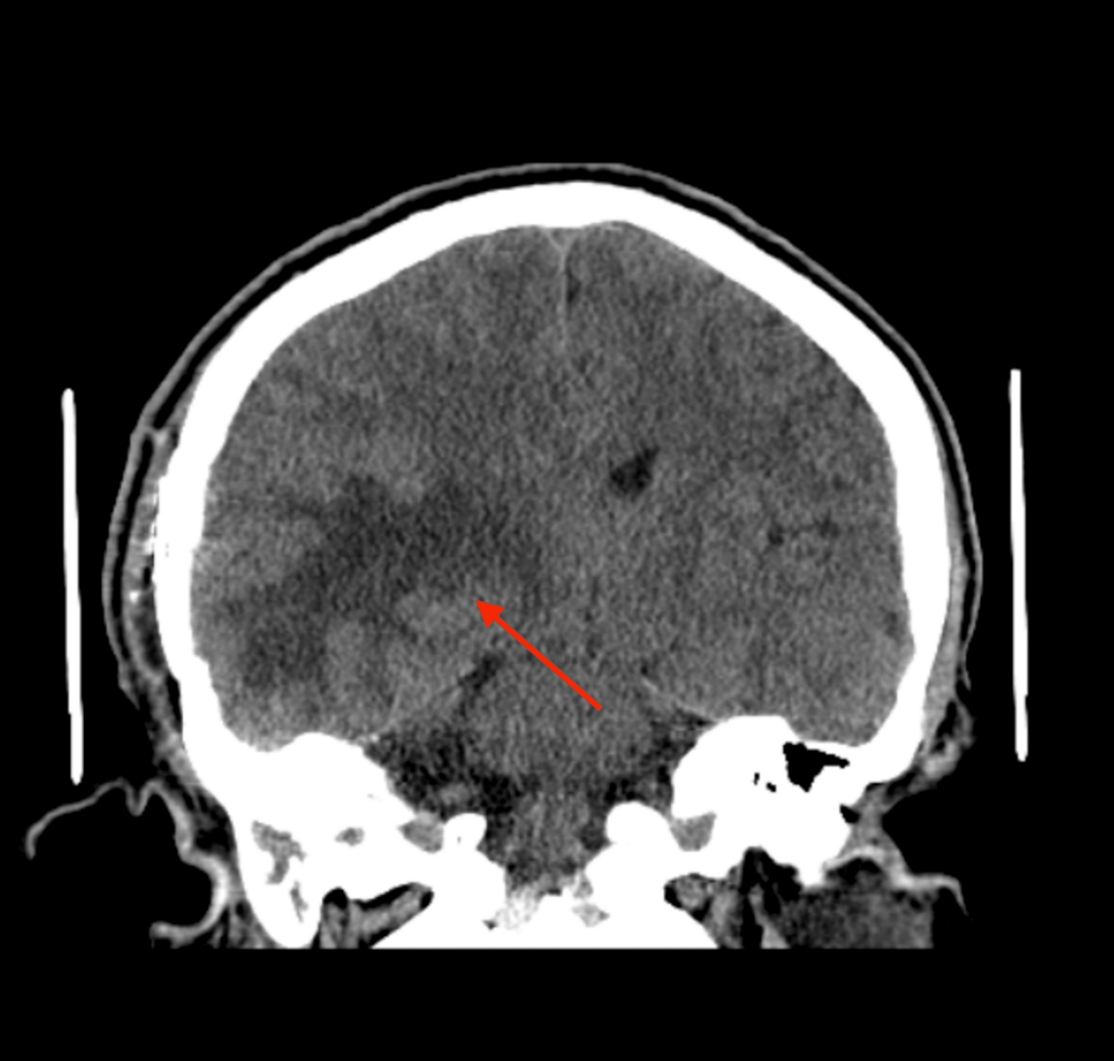
CT scan of the head (axial section) CT scan of the head (axial section) showing recurrent right temporal metastatic mass and 10 mm herniation to the left

**Figure 7:**
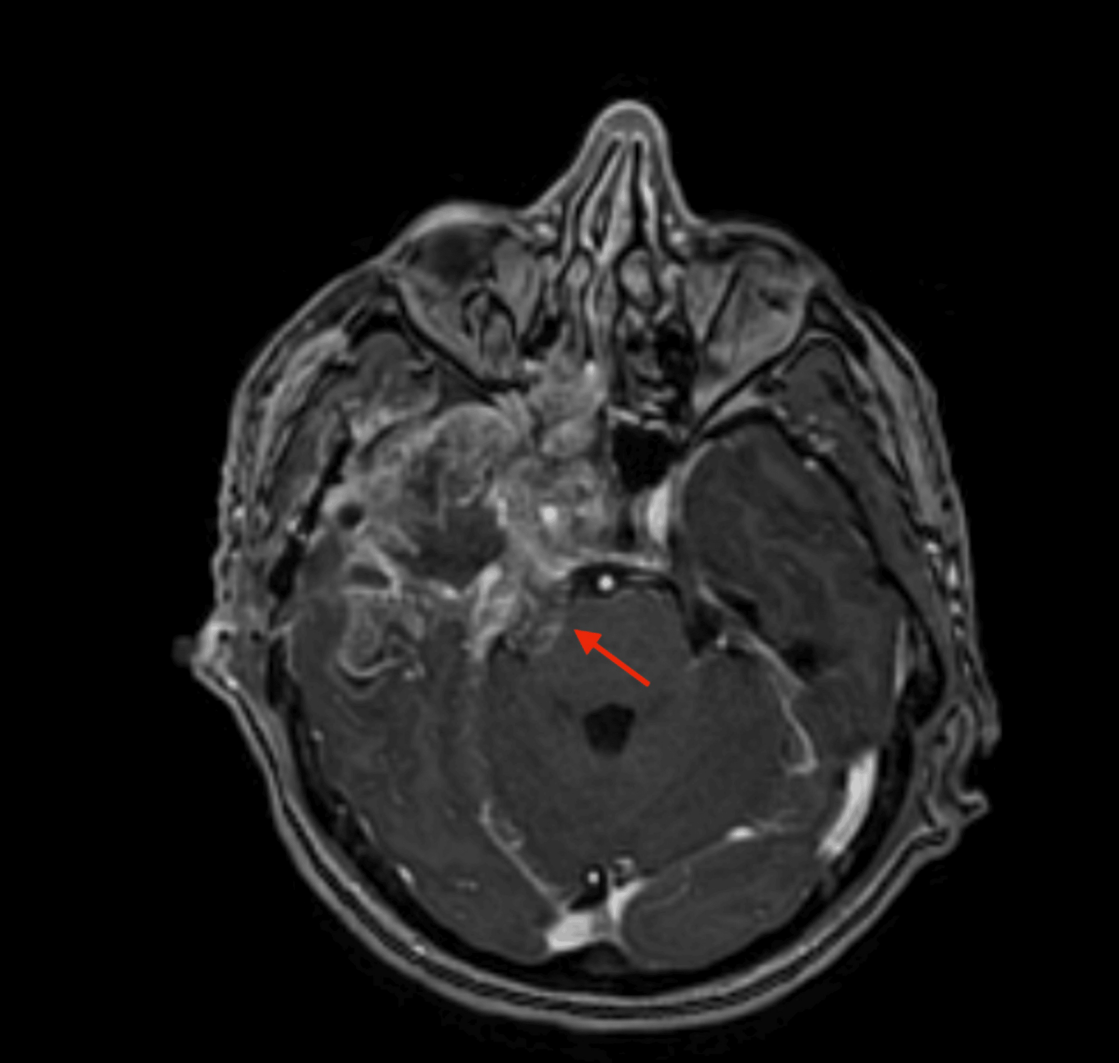
MRI of the brain The red line points at the tumor spread to the right cerebello-pontine angle.

**Figure 8:**
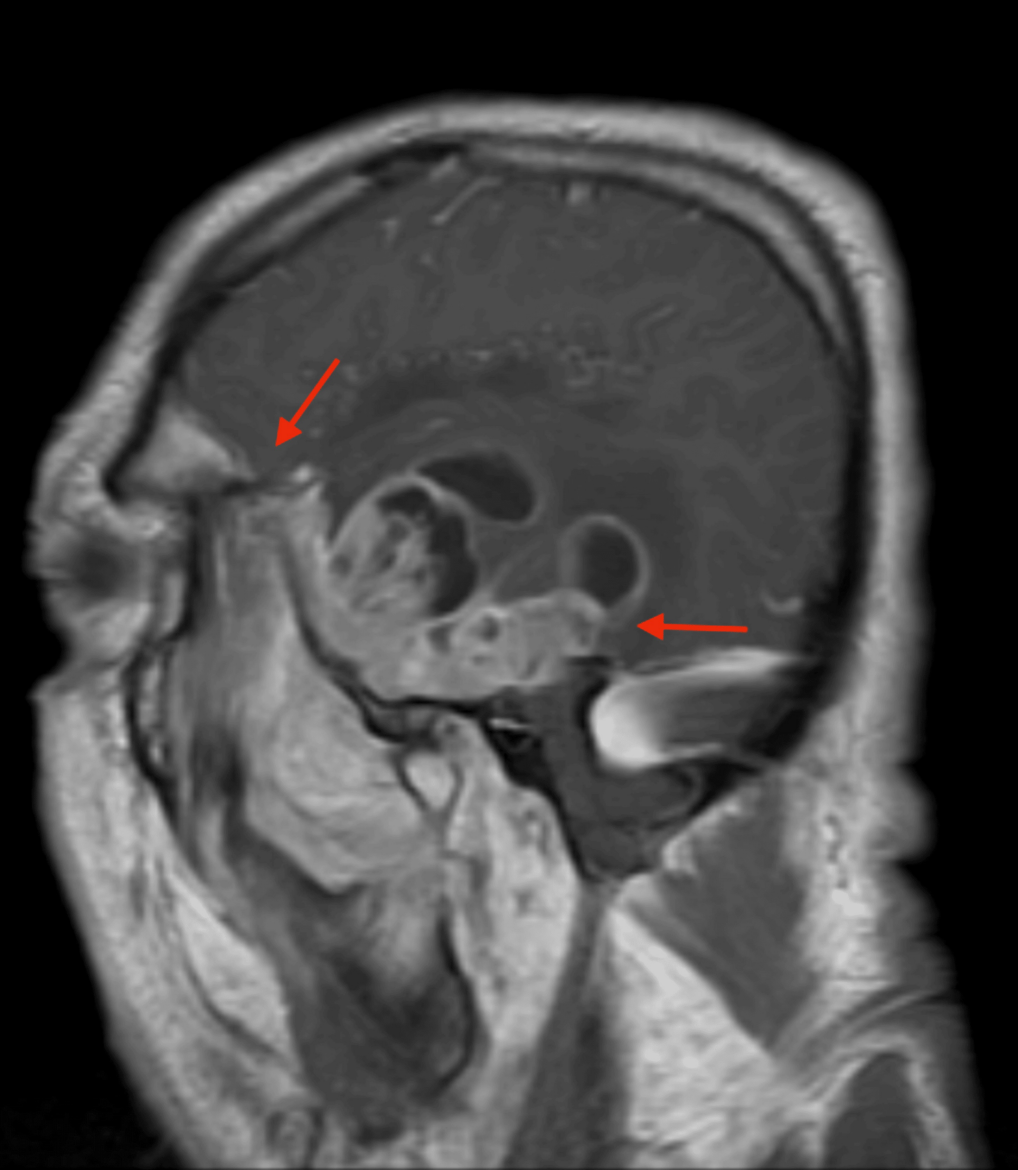
MRI of the brain MRI of the brain showed the right temporal lobe tumor extending into the sphenoid sinuses, the right cerebello-pontine angle, and the right retro-orbital region (red arrows).

**Figure 9:**
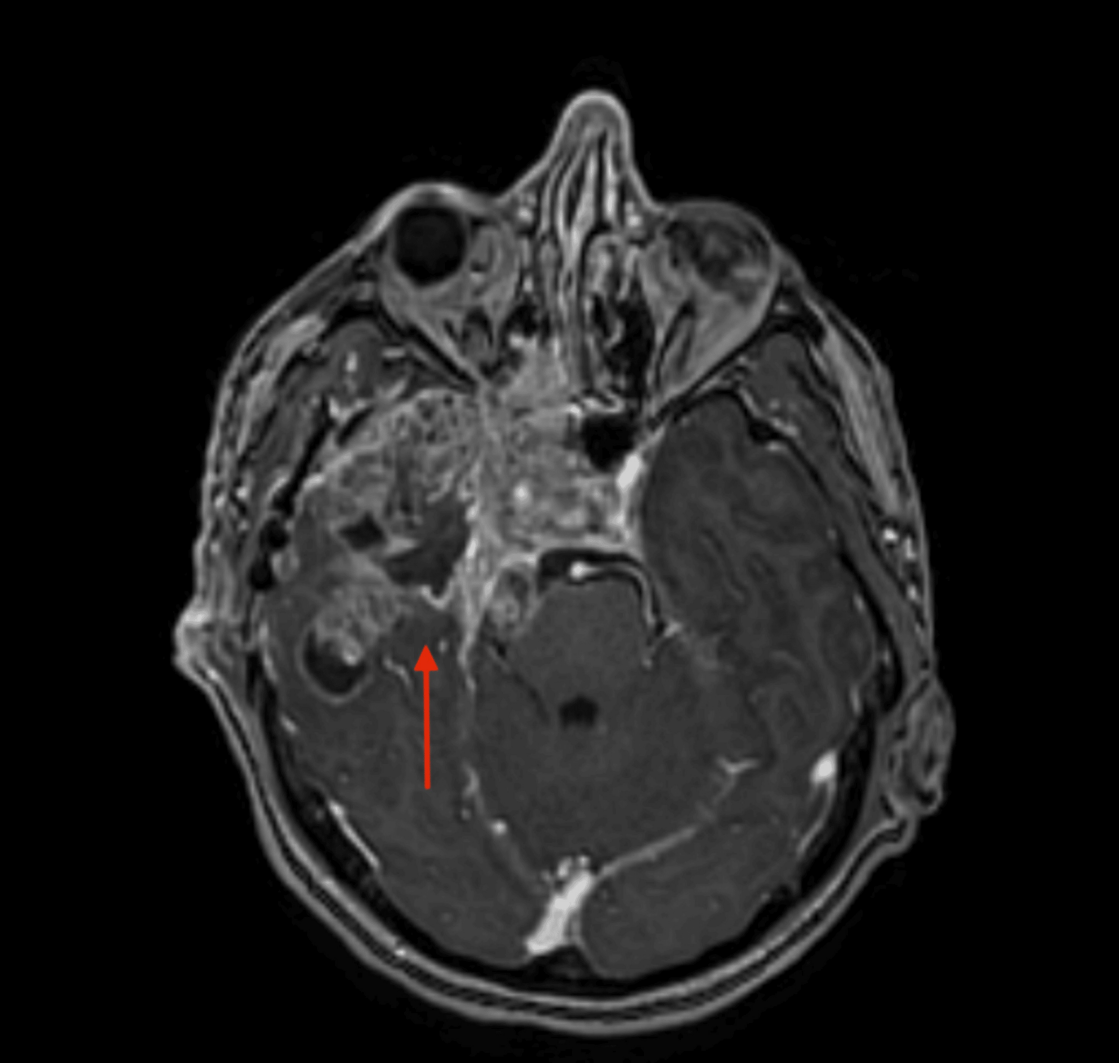
MRI of the brain MRI of the brain shows a large infiltrative tumor in the right temporal lobe extending into the right sphenoid sinus and right retro-orbital region, measuring approximately 6 x 8 cm.

Unfortunately, the patient did not follow up with his chemotherapy appointment. Two months later, the patient presented to the ED, complaining of an intolerable headache, difficulty walking, and facial droop. A CT scan of the head showed recurrent right temporal metastatic mass and 10 mm herniation to the left (Figure 6). MRI of the brain showed the right temporal lobe tumor extending into the sphenoid sinuses, right cerebellopontine angle, and right retro-orbital region (Figures 7-9). Neurosurgery recommended dexamethasone and levetiracetam, as the patient refused any invasive procedures, surgeries, chemotherapy, or radiation therapy. The resuscitation code was changed to do not resuscitate (DNR), as the patient preferred comfort care measures. 

## Discussion

Head and neck cancers usually commence in the squamous cell lining the mucosal surface of the head and neck (mouth, pharynx, and larynx); hence, they are referred to as head and neck squamous cell carcinomas (HNSCC), with less common origin from salivary glands or sinuses as well. Oral cavity cancers account for almost 50% of all HNSCCs. While multiple types of laryngeal tumors exist, squamous cell carcinomas represent more than 90% [7]. HNSCC in the larynx can arise from the vocal cords or epiglottis. The prevalence of HNSCC in the United States is approximately 4% of all cancers. Men are affected more than women. HNSCCs are more frequent around age 50 than in younger populations. More than 68,000 HNSCC cases are estimated in the US in 2021 [8].

Alcohol and tobacco use are the most common risk factors for developing HNSCC. Surprisingly, secondhand smoke and chewing tobacco can still provoke HNSCC [9]. Combined use of both alcohol and tobacco increases the likelihood of developing HNSCC compared to single-use [10]. Human papillomavirus (HPV), most commonly type 16, is a prevalent risk factor for HNSCC, mainly in the base of the tongue [11]. In June 2020, the FDA accelerated the approval of the HPV vaccine Gardasil 9 to prevent HNSCC caused by HPV types 16,18,31,33,45,52 and 58 for ages 9 through 45 years [12]. Symptoms of HNSCC usually precede awareness of a lump in the neck. Persistent sore throats, difficulty swallowing, and hoarseness in the voice are common. Laryngeal cancer often presents with a change in voice tone, difficulty breathing, talking, or swallowing, and/or ear pain.

The National Comprehensive Cancer Network (NCCN) recommends laryngeal SCC diagnosis through entire history taking and physical examination, a biopsy of the primary tumor site or fine needle aspiration of the neck, CT with contrast and thin angled cuts through the larynx, and magnetic resonance imaging (MRI) of the primary site and neck [13]. Treatment of HNSCC is individualized, depending on the location, stage, age, and other factors. The American Joint Committee on Cancer's TNM staging system for laryngeal SCC must be used for treatment options. Surgery and radiotherapy (RT) are two options for early-stage (T1-T2) glottic cancer, as the nodal disease is rare and the survival rates are high (90% for patients receiving RT, 93% for those undergoing transoral microsurgery) [14]. For advanced (stages III-IV), total laryngectomy with adjuvant RT is preferred over non-surgical therapy via chemotherapy. However, induction chemotherapy followed by RT has shown some clinical benefits.

HNSCCs spread locally to lymph nodes of the neck and very rarely spread distantly by hematogenous dissemination or, even rarer, through perineural invasion [15]. Brain metastasis [BM] has worse outcomes, with median survival ranging from 2.3 to 7.1 months [16]. Primary HNSCCs spread locally and have high rates of locoregional recurrences. Meanwhile, they can metastasize distantly in 10%-30% of cases, more commonly in the lung, liver, and bone, and extremely rarely to the brain [4]. BM can occur either secondary to hematogenous or perineural invasion of the tumor through seeding the intrafunicular spaces of the nerve that are in communication with subarachnoid spaces [17]. BM secondary to HNSCCs is more likely due to perineural invasion; therefore, any pain, paresis, or paresthesias in the distribution of cranial nerves should raise the alarm for BM in HNSCC patients. MRI can diagnose perineural invasion better than CT scans. BM secondary to HNSCC is difficult to treat, and the overall patient status must be considered before planning treatment. The gold standard treatment for HNSCC with BM includes surgical excision with or without post-operative whole-brain radiation; meanwhile, stereotactic radiosurgery may play a role. It is recommended to start adjuvant radiation therapy after surgery in cases of evident perineural invasion on MRI.

The survival rate for laryngeal cancer has improved significantly over the years; however, recurrence is reported in 22%-31% after 2-3 years of treatment completion [18]. The high risk of recurrence in laryngeal cancers includes advanced stages, a positive <1 mm or close resection margin (1-5 mm), vascular or lymphatic invasion, extranodal extension, and perineural invasion. In those cases, adjuvant treatment is recommended [19]. In June 2023, Shin et al. conducted a retrospective study to assess perineural invasion in HNSCC as a prognostic factor. They studied 240 patients with laryngeal SCC and concluded that perineural invasion, independent of treatment history, is a statistically significant prognostic factor associated with a poor survival rate and local recurrence in laryngeal cancer. In 2018, Montano et al. conducted the first study to analyze the survival of BM from laryngeal SCC [20]. According to the study, only 18 cases were reported as BM with laryngeal SCC primary origin. Most of the metastases were in the cavernous sinus, followed by the pituitary gland, and only one case had multiple BM and concomitant meningeal metastases. They found that surgery and/or radiochemotherapy increased survival compared to non-treated cases.

## Conclusions

Laryngeal cancer is a very common head and neck malignant neoplasm. Laryngeal cancer can be glottic, supraglottic, or subglottic tumors. Smoking and alcohol are the two most common risk factors for laryngeal cancers, and high-risk HPV can also induce laryngeal cancer. Laryngeal cancer tends to have a direct invasion and is less likely to spread distantly. Perineural invasion is a poor prognostic factor and is more associated with locoregional metastasis to the brain. Surgery and adjuvant radiotherapy are the main lines of treatment, and chemotherapy has been growing recently. Our case represents a sporadic behavior for LSCC with locoregional recurrence in the brain. The patient's brain metastasis was a perineural invasion of the laryngeal mass rather than a distant metastasis. Very little literature has discussed the management of laryngeal cancer with perineural invasion of the brain and the recurrence of brain metastasis even after excision. 
